# European Safe Community Network

**Published:** 2019-08

**Authors:** Mirjana Milankov, Miodrag Blizanac, Jovan Vignjevic, Mila Radovanovic

**Affiliations:** ^*a*^National center for injury prevention and safety promotion, Novi Sad, Serbia.; ^*b*^NGO Faith, Love, Hope, Novi Sad, Serbia.

## Abstract

More than 5 million people die each year as a result of: injuries, resulting from acts of violence against oneself or others, road traffic crashes, burns, drowning, falls, poisonings, among other causes. That is the reason for Safe Community program in every part of the world. Because Safe Communities are an Essential need for a Modern-Day Society. Safe Community is our present and future. Safe Community does not know the state boundaries, nationality, religion, skin color, age and gender. Safe Community is a global program and global movement for the preservation and protection of the environment, life and health of every inhabitant of this planet. Safe Community is a safe environment in which people can develop all their work and productive potentials. European safe Community Network aims at strengthening existing International Safe Communities in Europe and promotion of the formation of new communities to be accepted into the Safe community movement. The Global Burden of Disease Studies has documented unfair distribution of injury by socioeconomic strata in Europe. This gradient, existing within and between countries, must be addressed. Our board has representatives from Novi Sad in South Eastern Europe through the Baltics and as far north in Scandinavia as 200 kilometers north of the Arctic Circle in Norway. Together we are working against public indifference towards the burden of injury and trying to promote community based interventions against injuries e.g. traffic accidents , drowning, burns, falls, violence, and suicide. In the 53 countries of the WHO European Region injuries are estimated to cause 790 000 deaths yearly. This is 9% of deaths from all causes. An estimate for the 27 countries in the European Union is 252 000 injury related deaths and about 7 million hospital admissions yearly (8% of all admissions). Injury is the greatest thief of life years in the age group from one year to the mid-forties. The first Safe Community was designated in 1989. Several Swedish and Norwegian communities followed in Falkopings, footsteps during the next two decades. There are 58 designated Safe Communities in Europe. Of these 36 are in Sweden and Norway. Norway has a strong National Safe Community Network, Norwegian Safety Forum and are steadily recruiting communities up to the present. Sweden he s recruited few new safe communities the last two decades, probably due to the dissolving of the Swedish National Network. However, existing Swedish Safe Communities are active and strong and thus redesigned. Key success on participation in National and International Safe Communities Networks are: Statistics, Research, Strategy, Motivation, Education, Training and Collaboration.

**Keywords:**

Safe Community, European Network, Statistics, Research, Strategy, Motivation, Education, Training and Collaboration

